# Author Correction: Small molecule inhibition of Dynamin-dependent endocytosis targets multiple niche signals and impairs leukemia stem cells

**DOI:** 10.1038/s41467-021-21688-1

**Published:** 2021-02-19

**Authors:** Cedric S. Tremblay, Sung Kai Chiu, Jesslyn Saw, Hannah McCalmont, Veronique Litalien, Jacqueline Boyle, Stefan E. Sonderegger, Ngoc Chau, Kathryn Evans, Loretta Cerruti, Jessica M. Salmon, Adam McCluskey, Richard B. Lock, Phillip J. Robinson, Stephen M. Jane, David J. Curtis

**Affiliations:** 1 Australian Centre for Blood Diseases, Central Clinical School, Monash University, Melbourne, VIC Australia; 2 Department of Clinical Haematology, Alfred Health, Melbourne, VIC Australia; 3 Lowy Cancer Research Centre, Children’s Cancer Institute, University of New South Wales, Sydney, NSW Australia; 4 Cell Signalling Unit, Children’s Medical Research Institute, Sydney, NSW Australia; 5 Chemistry, Centre for Chemical Biology, School of Environmental and Life Sciences, University of Newcastle, Callaghan, NSW Australia

**Keywords:** Acute lymphocytic leukaemia, Acute myeloid leukaemia, Growth factor signalling, Endocytosis, Cancer stem cells

Correction to: *Nature Communications* 10.1038/s41467-020-20091-6, published online 4 December 2020.

In the original version of this Article, contributions from Jennifer R. Baker and Cecilia C. Russell from the University of Newcastle for synthesizing batches of Dynole 34-2 were not acknowledged. This error has now been corrected in both the PDF and HTML versions of the Article.

