# Supplementary material for: Small molecule inhibition of Dynamin-dependent endocytosis targets multiple niche signals and impairs leukemia stem cells
Source: Nat Commun. 2020 Dec 4;11:6211. doi: 10.1038/s41467-020-20091-6 (PMC7719179; doi:10.1038/s41467-020-20091-6)
Supplement: Supplementary file 1 — Supplementary Information [file 41467_2020_20091_MOESM1_ESM.pdf]

Small molecule inhibition of Dynamin-dependent endocytosis targets multiple niche signals and impairs leukemia stem cells

Tremblay CS, *et al*.

SUPPLEMENTARY FIGURES…………………………………………...………..…………2

Supplementary Figure 1. *In vitro* treatment of BaF3-IL7R cells with Dynamin inhibitors, Ruxolitinib and Vincristine…………………………………………………………………………………..……2

Supplementary Figure 2. *In vitro* efficacy of Dynole 34-2 on hematopoietic cells……..………..……4

Supplementary Figure 3. *In vivo* effect of Dynole 34-2 ………………………………………………..6

Supplementary Figure 4. Dynole 34-2 sensitizes pre-LSCs to induction-like therapy……………..….8

Supplementary Figure 5. Dynole 34-2 has no detrimental effect on normal hematopoiesis ……..….10

Supplementary Figure 6. Efficacy of Dynole 34-2 on primary *Lmo2*-transgenic T-ALL……….…....12

Supplementary Figure 7. Efficacy of Dynole 34-2 for human T-ALL...……………….……………..…14

Supplementary Figure 8. *In vitro* treatment of BaF3-SGM3R cells with Dynole 34-2……..…...…16

Supplementary Figure 9. Efficacy of Dynole 34-2 for human AML...……………………………..…18

Supplementary Figure 10. Dynamin inhibitors target relapse-inducing cells in acute leukemia....…20

Supplementary Figure 11. Gating strategies for flow cytometric analyses.................................…21

SUPPLEMENTARY REFERENCES……………………………………...………..………22

Supplementary Figures

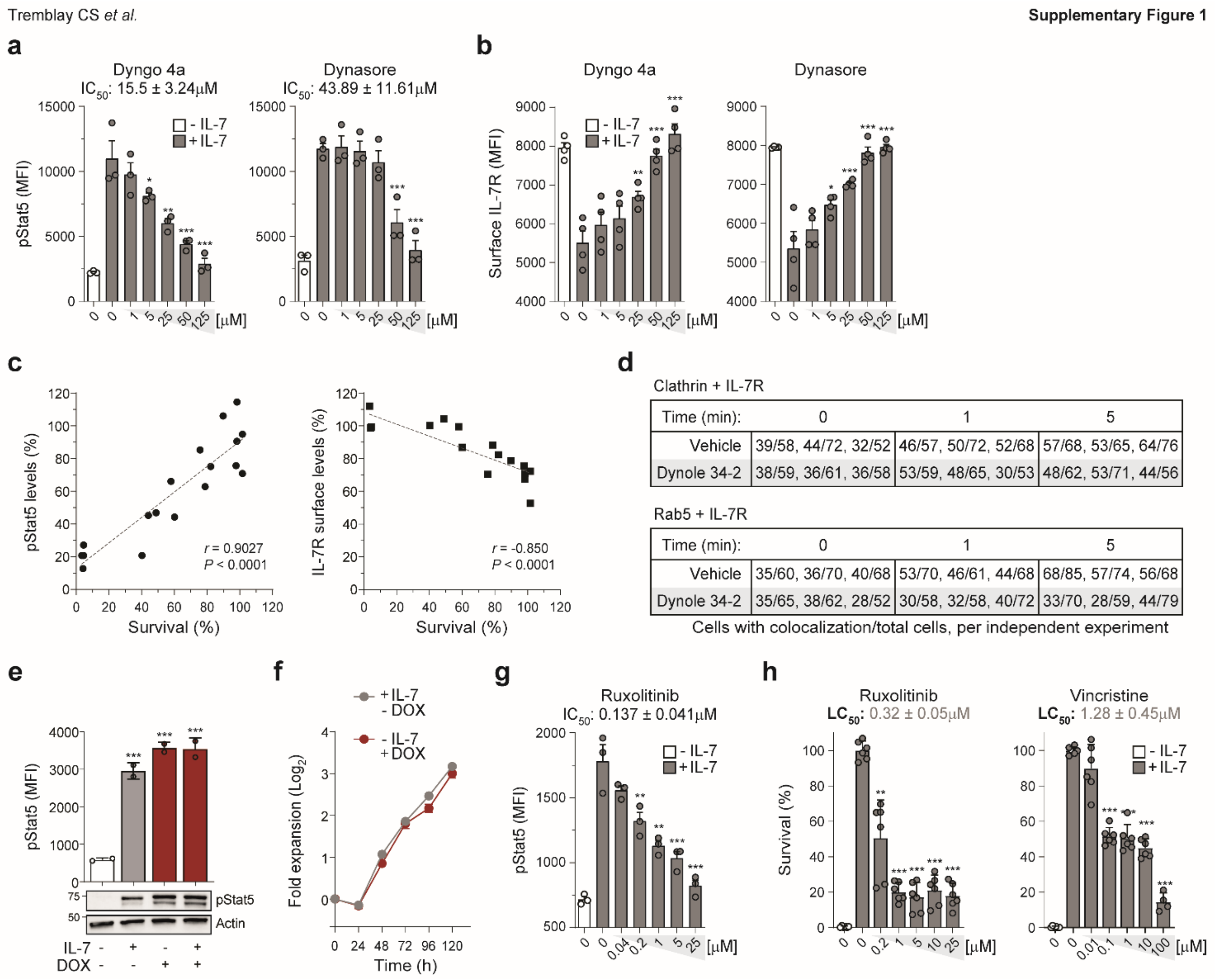

Supplementary Figure 1. *In vitro* treatment of BaF3-IL7R cells with Dynamin inhibitors, Ruxolitinib and Vincristine. a, Levels of activated Stat5 (pStat5) in BaF3-IL7R cells treated with increasing doses of Dyngo 4a and Dynasore assessed by flow cytometry. Mean fluorescence intensity (MFI) ± SD of n=3 technical replicates, performed in 3 independent experiments (*P<0.05, **P<0.01, ***P<0.001 compared to vehicle + IL-7; grey). Basal levels of pStat5 were measured in unstimulated BaF3-IL7R cells (-IL-7; white bar). Median inhibitory concentration (IC_{50}) of Dyngo 4a and Dynasore on pStat5 is indicated. **b,** Surface expression of IL-7 receptor (IL-7R) treated with Dyngo 4a and Dynasore assessed by flow cytometry. MFI ± SD of n=4 technical replicates, performed in 2 independent experiments (*P<0.05, **P<0.01, ***P<0.001 compared to vehicle + IL-7; grey). Baseline measured in unstimulated BaF3-IL7R cells (-IL-7; white bar). **c,** Correlative studies between the relative levels of pStat5 (left) and IL-7R surface levels (right) with the relative survival

of BaF3-IL7R cells treated Dynole 34-2. Pearson correlation coefficient *r* is indicated. Student's *t*-test. Levels of pStat5, IL-7R and viability were normalized to cells treated with vehicle (%DMSO). **d,** Dynamin colocalization of IL-7R with Clathrin (top) and Rab5 (bottom) in Ba/F3-IL-7R cells at different timepoints following IL-7 stimulation, in the presence of either Dynole 34-2. Total number of cells assessed and displaying colocalization are indicated (n=3 independent experiments). **e,** Levels of pStat5 in BaF3-IL7R$^{Stat5\text{-}CA}$ cells cultured in the presence or absence of IL-7 (grey) and doxycycline (red) assessed by flow cytometry (top) and Western blots (bottom). MFI ± SD of n=2 technical replicates, performed in 2 independent experiments (***P<0.001 compared to unstimulated cells; white bar). For Western blots, Actin was used as a loading control. **f,** Proliferation of BaF3-IL7R$^{Stat5\text{-}CA}$ cells in absence (+IL-7 –DOX; grey) or presence (-IL-7 +DOX; red) of doxycycline. Fold expansion was calculated from the number of cells initially put into culture at day 0. Mean ± SD (n=3). **g,** Levels of pStat5 in BaF3-IL7R cells treated with Ruxolitinib. Basal levels of pStat5 were measured in unstimulated cells (-IL-7, white bar). Median inhibitory concentration (IC_{50}) of Ruxolitinib on IL-7—induced Stat5 activation (pStat5) is indicated. MFI ± SD of n=3 technical replicates, performed in 3 independent experiments (**P<0.01, ***P<0.001 compared to vehicle + IL-7). **h,** Relative Survival of IL-7—dependent (+IL-7; grey) BaF3-IL7R cells treated with Ruxolitinib or Vincristine for 48 hours. Viability was normalized to cells treated with vehicle (%DMSO). Median lethal concentration (LC_{50}) of each drug tested is indicated. Mean ± SD of n=6 technical replicates, performed in 3 independent experiments. Student's *t*-test **P<0.01, ***P<0.001 compared to vehicle. Basal levels of live cells were measured in BaF3-IL7R cells cultured without cytokines (-IL-7; white bar).

a

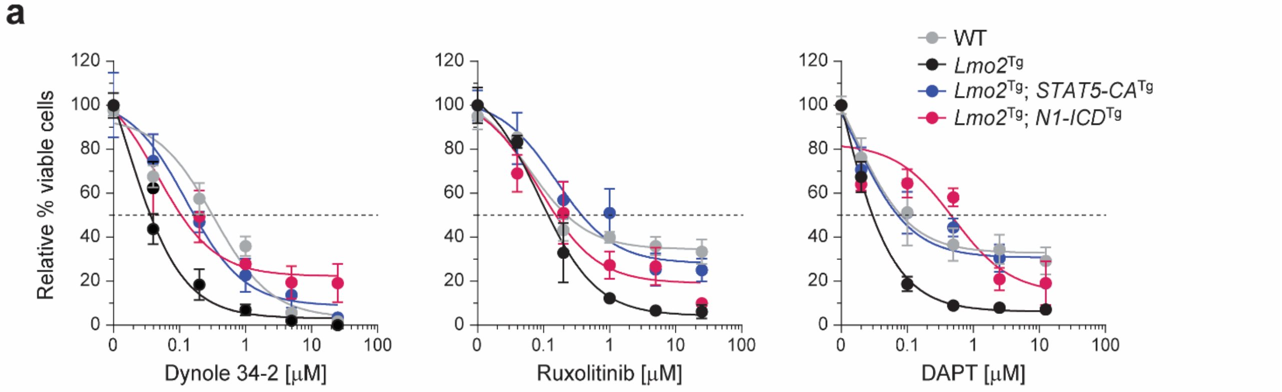

b

Genotypes	LC50 [μM] Dynole 34-2	Ruxolitinib	DAPT
WT	0.286 ± 0.050	0.342 ± 0.137	0.188 ± 0.065
Lmo2^{Tg}	*** 0.054 ± 0.011	* 0.139 ± 0.034	*** 0.045 ± 0.008
Lmo2^{Tg}; *STAT5-CA*^{Tg}	# ** 0.189 ± 0.050	## 0.741 ± 0.249	** 0.208 ± 0.074
Lmo2^{Tg}; *N1-ICD*^{Tg}	## * 0.185 ± 0.058	0.225 ± 0.070	### 0.404 ± 0.136

c

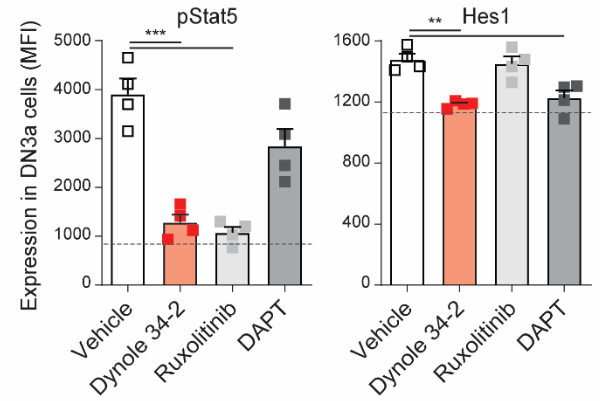

d **e**

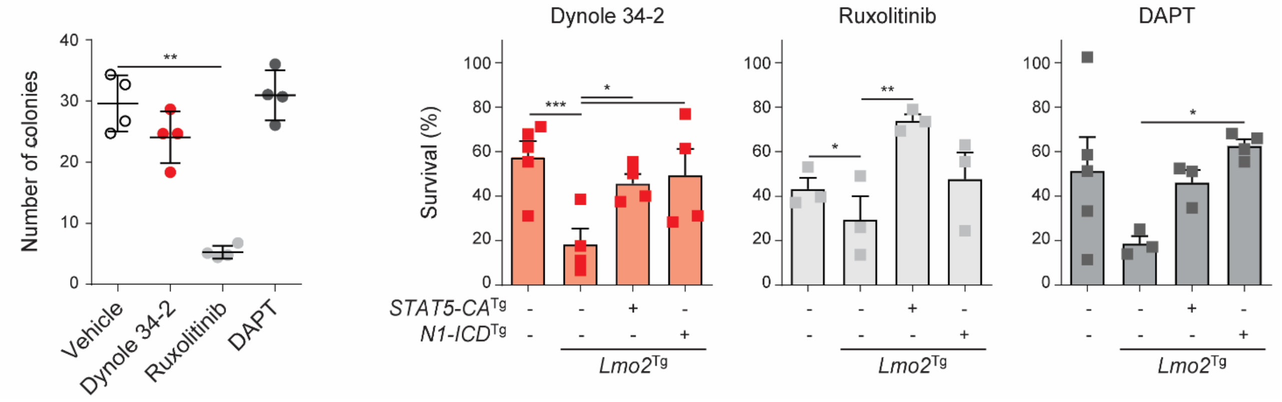

Supplementary Figure 2. *In vitro* efficacy of Dynole 34-2 on hematopoietic cells. a, Relative viability of wild-type (WT), *Lmo2*^{Tg}, *Lmo2*^{Tg};*STAT5-CA*^{Tg} and *Lmo2*^{Tg};*N1-ICD*^{Tg} DN3a thymocytes treated with increasing concentration of Dynole 34-2, Ruxolitinib or DAPT for 48 hours. Viability was normalized to cells treated with vehicle (%DMSO). Mean ± SEM of n=4 biological replicates. **b,** Median lethal concentration (LC_{50}) of Dynole 34-2, Ruxolitinib and DAPT assessed on DN3a thymocytes from 6-week old wild-type (WT), *Lmo2*^{Tg}, *Lmo2*^{Tg};*STAT5-CA*^{Tg} and *Lmo2*^{Tg} ;*N1-ICD*^{Tg} mice. Mean ± SD of n=4 biological replicates are shown. 2-way ANOVA with Tukey's correction test;

*P<0.05, ***P<0.001 compared to WT; #P<0.05, ##P<0.01, ###P<0.001 compared to *Lmo2*^{Tg}. **c,** Levels of pStat5 (left) after stimulation with IL-7, as well as Hes1 expression (right) after co-culture on OP9-DL1 cells overnight, in *Lmo2*-transgenic DN3a thymocytes treated with either vehicle, Dynole 34-2 (0.2 μM), Ruxolitinib (0.2 μM) and DAPT (0.1 μM). Unstimulated cells were used as negative controls (dashed line). Student's *t*-test, **P<0.01, ***P<0.001 compared to unstimulated cells. MFI ± SEM, 1-way ANOVA with Tukey's correction test; **P<0.01, ***P<0.001 compared to vehicle. **d**, Clonogenic assay of normal murine hematopoietic stem and progenitor cells (HSPCs) cultured in the presence of either vehicle, Dynole 34-2 (0.2 μM), Ruxolitinib (0.2 μM) and DAPT (0.1 μM) for 10 days. Mean ± SD, Student's *t*-test (n=4) **P<0.01 compared to vehicle. **e,** Relative viability of wild-type (WT), *Lmo2*^{Tg}, *Lmo2*^{Tg};*STAT5-CA*^{Tg} and *Lmo2*^{Tg};*N1-ICD*^{Tg} DN3a thymocytes treated with either Dynole 34-2 (0.2 μM), Ruxolitinib (0.2 μM) and DAPT (0.1 μM) for 48 hours. Viability was normalized to cells treated with vehicle (%DMSO, which was reported as 100). Mean ± SEM, 2-way ANOVA with Tukey's correction test, **P<0.01, ***P<0.001 compared to vehicle.

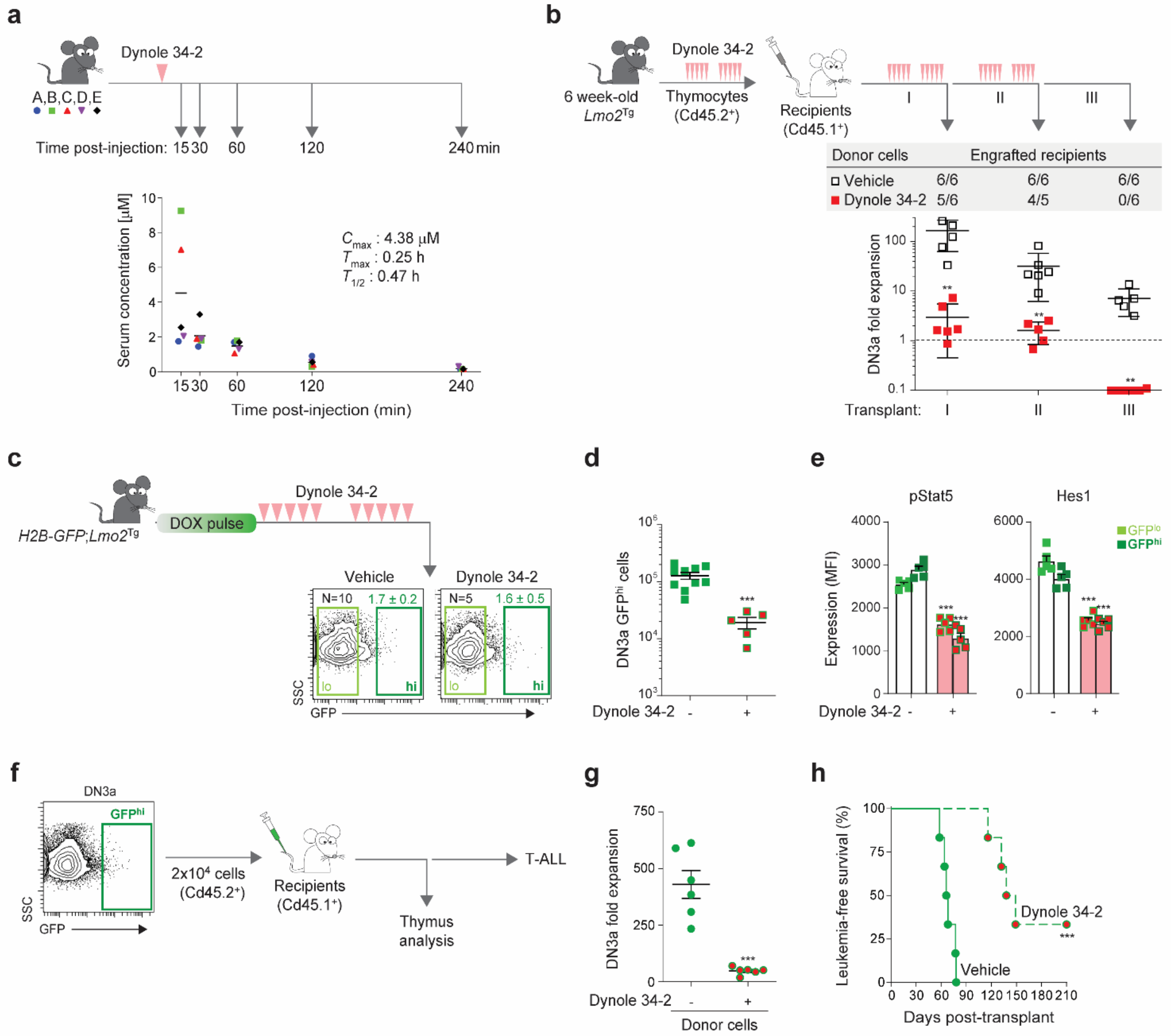

Supplementary Figure 3. *In vivo* effect of Dynole 34-2. a, Treatment schematic and pharmacokinetic analysis of serum levels (µM) of Dynole 34-2 at different time points (15, 30, 60, 120 and 240 minutes) following a single intraperitoneal injection (grey arrow) in 5 recipients (A, B, C, D, E). Average maximum concentration (C_{max}), the time taken to reach this maximum concentration (T_{max}), and the half-life ($T_{1/2}$) of Dynole 34-2 in the serum of treated mice are indicated. Average concentration for each time point is indicated (bar). **b,** Treatment schematic and fold expansion of donor-derived *Lmo2*-transgenic DN3a thymocytes enumerated in the thymus of primary (I), secondary (II) and tertiary (III) recipients, assessed four weeks after transplantation of cells harvested from 5 independent mice per cohort of donors. Number of recipients engrafted for each condition is indicated. Mean ± SD (N = number of independent mice analysed), Student's *t*-test;

$P<0.01$. **c, Scheme for GFP labelling followed by 2 weeks of chase without Doxycycline, with representative flow cytometric analysis of GFP expression in DN3a thymocytes from *H2B-GFP;Lmo2*^{Tg} mice at 24 hours following the last administration of Dynole 34-2 or vehicle. GFP^{lo} and GFP^{hi} populations are framed, with the average proportion (mean ± SD) of GFP^{hi} cells indicated, Student's *t*-test, as compared to the control. **d,** Absolute numbers of DN3a GFP^{hi} cells in the thymus of from *H2B-GFP;Lmo2*^{Tg} mice at analysis. Student's *t*-test; ***$P<0.001$. **e,** Levels of pStat5 (left) and Hes1 expression (right) in GFP^{lo} and GFP^{hi} populations of DN3a cells analysed in the thymus of *H2B-GFP;Lmo2*^{Tg} mice administered with Dynole 34-2 or vehicle. MFI ± SEM (n=5 individual mice). 1-way ANOVA with Tukey's correction test; ***$P<0.001$ compared to vehicle. **f,** Scheme for transplantation assays of $2x10^4$ purified DN3a thymocytes from *H2B-GFP;Lmo2*^{Tg} mice into sublethally-irradiated isogenic (Cd45.1$^+$) recipients. The thymus of a cohort of recipients was harvested for analysis 6 weeks after transplantation, whereas another cohort was monitored for leukemia development. **g,** Fold expansion of donor-derived DN3a cells in the thymus of recipients injected with GFP^{hi} DN3a thymocytes harvested from *H2B-GFP;Lmo2*^{Tg} mice treated with Dynole 34-2 or vehicle. Mean ± SD, Student's *t*-test; ***$P<0.001$. **h,** Kaplan-Meier curves of the time to leukemia for recipients injected with purified GFP^{hi} DN3a cells from the thymus of *H2B-GFP;Lmo2*^{Tg} mice treated with Dynole 34-2 (N=6) or vehicle (N=6). Log-rank (Mantel-Cox) test; ***$P<0.001$ compared to vehicle. All malignant thymic tumors were diagnosed at necropsy.

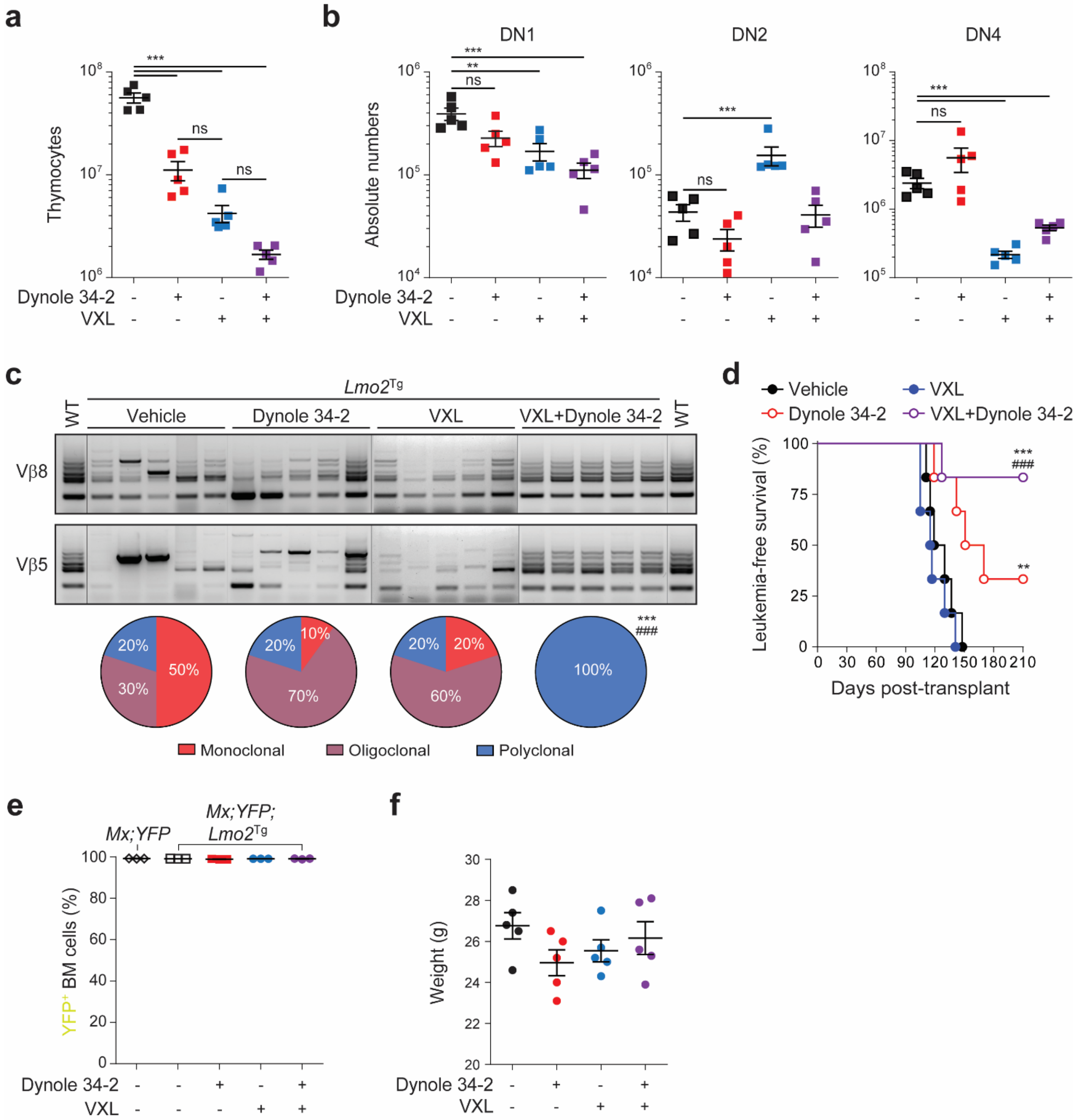

Supplementary Figure 4. Dynole 34-2 sensitizes pre-LSCs to induction-like therapy. a-b, Absolute number of thymocytes (**a**), as well as immature populations (**b**) in the thymus of *Lmo2*Tg mice, assessed by flow cytometry 24 hours after the last dose of either Dynole 34-2, induction-like therapy (VXL) or combination therapy was administered. Mean ± SEM, 2-way ANOVA with Tukey's correction test; ***P*<0.01, ****P*<0.0001 compared to vehicle. **c,** *Tcrβ* gene rearrangement analysis in purified DN3a thymocytes from 2-month old *Lmo2*Tg mice (panel) and proportion of mono-, oligo- and poly-clonal *Tcrβ* gene rearrangements (pie charts) in these purified thymocytes (n = 5). Two-way

ANOVA test with a Two-stage linear step-up procedure of Benjamin, Krieger and Yekutieli; ****P*<0.001 compared to vehicle; ###*P*<0.001 compared to VXL. **d,** Kaplan-Meier curves of the time to leukemia for recipients injected with thymocytes harvested from *Lmo2*^{Tg} mice treated Dynole 34-2, VXL and combined therapy, 24 hours after the last dose was administered. Log-rank (Mantel-Cox) test (N=6 per cohort); ***P*<0.01, ****P*<0.001 as compared to vehicle; ###*P*<0.001 compared to VXL. All malignant thymic tumors were diagnosed at necropsy. **e**, Proportion of HSC-derived cells (YFP^{+}) in the bone marrow (BM) of *Mx*;*YFP;Lmo2*^{Tg} mice treated with vehicle, Dynole 34-2, VXL and the combination Dynole 34-2 + VXL. *Mx*;*YFP* mice were used as positive controls. Mean ± SEM, 2-way ANOVA with Tukey's correction test. **f**, Weight of the mice from cohorts treated with vehicle, Dynole 34-2, VXL and the combination therapy, 24hours after the last administration.

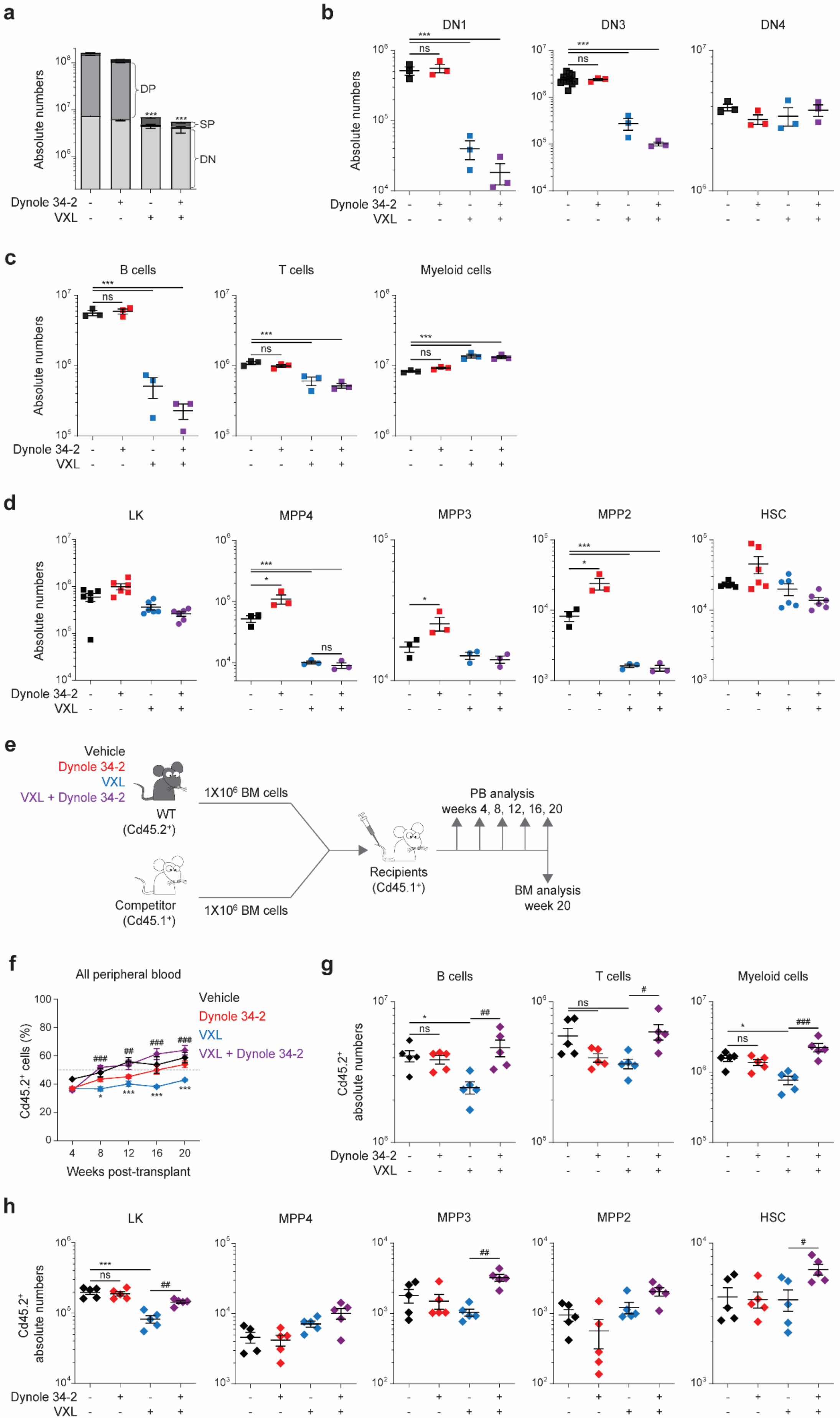
a
Absolute numbers
DP
SP
DN
Dynole 34-2
VXL
b
DN1
DN3
DN4
c
B cells
T cells
Myeloid cells
d
LK
MPP4
MPP3
MPP2
HSC
e
Vehicle
Dynole 34-2
VXL
VXL + Dynole 34-2
WT
(Cd45.2+)
1X10^6 BM cells
Competitor
(Cd45.1+)
Recipients
(Cd45.1+)
PB analysis
weeks 4, 8, 12, 16, 20
BM analysis
week 20
f
All peripheral blood
Cd45.2+ cells (%)
Weeks post-transplant
g
Cd45.2+ absolute numbers
h

Supplementary Figure 5. Dynole 34-2 has no detrimental effect on normal hematopoiesis. a-b, Absolute number of thymocytes (**a**), as well as immature populations (**b**) in the thymus of wild-type (WT) mice, assessed by flow cytometry 24 hours after the last dose of either Dynole 34-2, induction-like therapy (VXL) or combination therapy was administered. Mean ± SEM, 2-way ANOVA with Tukey's correction test; $^{**}P<0.01$, $^{***}P<0.0001$ compared to vehicle. **c-d,** Absolute numbers of murine B ($CD19^+$ $B220^+$), T ($CD3^+$) and Myeloid (Gr-1^+ $CD11b^+$ granulocytes and monocytes) cells (**c**), as well as Lineage-negative Kit-positive (Lin^-Kit^+, LK) progenitors, different populations of multipotent progenitors (MPP) and hematopoietic stem cells (HSC; **d**) from the bone marrow of WT mice treated with Dynole 34-2, VXL and combined therapy, 24 hours after the last dose was administered. Vehicle was used as control. **e,** Experimental schematic of competitive transplant using $1x10^6$ cells from WT mice treated with vehicle, Dynole 34-2, VXL and combined therapy, harvested 24 hours after the last dose was administered, mixed in a 1:1 proportion with BM cells harvested from isogenic $Cd45.1^+$ mice. These cells were injected into lethally-irradiated recipients, and lineage reconstitution was monitored every 4 weeks, until 20 weeks post-transplantation when BM was harvested for analysis. **f**, Percent of donor-derived ($Cd45.2^+$) cells among all peripheral blood cells at 4, 8, 12, 16 and 20 weeks post-transplant. **g-h**, Absolute numbers of donor-derived ($Cd45.2^+$) lineage-committed (B, and Myeloid) cells (**g**), as well as LK, MPP populations and HSCs (**h**) harvested from the BM of recipients at 20 weeks post-transplant. Mean ± SEM, 2-way ANOVA with Tukey's correction test; $^{*}P<0.05$, $^{***}P<0.001$ compared to vehicle; $^{\#}P<0.05$, $^{\#\#}P<0.01$, $^{\#\#\#}P<0.001$ compared to VXL. ns = not statistically significant.

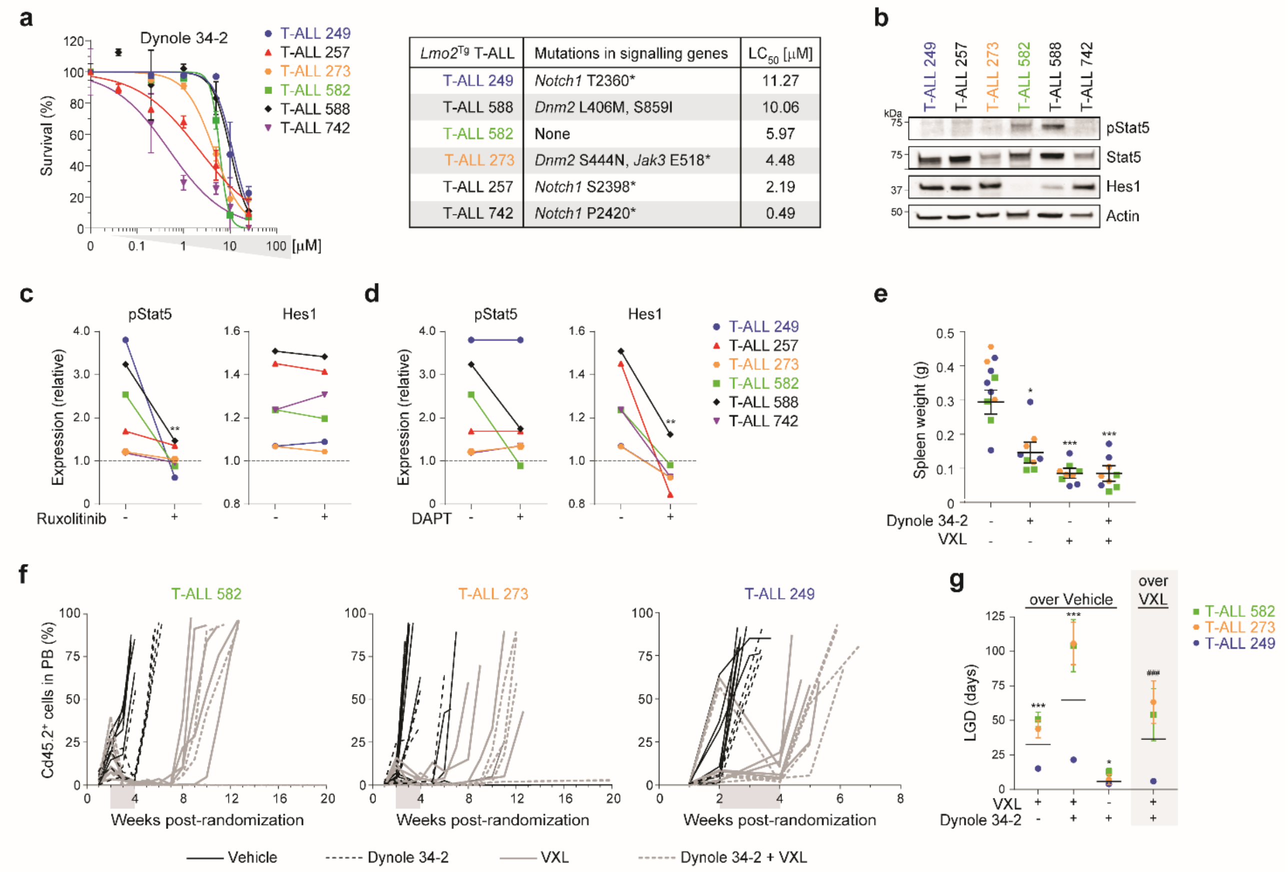

$Lmo2^{Tg}$ T-ALL	Mutations in signalling genes	LC_{50} [µM]
T-ALL 249	*Notch1* T2360*	11.27
T-ALL 588	*Dnm2* L406M, S859I	10.06
T-ALL 582	None	5.97
T-ALL 273	*Dnm2* S444N, *Jak3* E518*	4.48
T-ALL 257	*Notch1* S2398*	2.19
T-ALL 742	*Notch1* P2420*	0.49

Supplementary Figure 6. Efficacy of Dynole 34-2 on primary *Lmo2*-transgenic T-ALL. a, Relative viability (%) of primary *Lmo2*^{Tg} leukemias treated with increasing concentration of Dynole 34-2 for 48 hours. Viability was normalized to vehicle-treated cells with experiments performed in duplicates or triplicates. Mutations of growth factor-induced signalling pathways and median lethal concentration (LC_{50}) of Dynole 34-2 for each primary *Lmo2*^{Tg} T-ALL are indicated (right panel). The amino-acid position for each mutation is shown, * means frameshift. **b,** Levels of activated Stat5 (pStat5) and Hes1 determined by Western blot. Stat5 and Actin were used as loading controls. **c, d,** Relative levels of pStat5 and Hes1 in DN3a cells from primary *Lmo2*^{Tg} T-ALL treated *in vitro* with Ruxolitinib (**c**) or DAPT (**d**), after stimulation with either IL-7 (pStat5, left) or DL1 (Hes1, right). Primary leukemias are indicated on the right; unstimulated leukemic DN3a cells treated with vehicle were used as control, and reported as 1 (dashed line). Mean ± SEM, 2-way ANOVA with Bonferroni correction test; **P<0.01 as compared to vehicle. **e**, Spleen weight in recipients 24 hours after the last administered dose of Dynole 34-2, VXL and combination therapy. Mean ± SEM, 2-way ANOVA with Bonferroni correction test; *P<0.05, ***P<0.001 as compared to vehicle. **f,** Proportion of donor-

derived leukemic cells (%Cd45.2^{+}) in the peripheral blood (PB) of recipients injected with *Lmo2*^{Tg} primary leukemias (T-ALL 582, n=5/cohort; T-ALL 273, n=7/cohort; T-ALL 249, n=5/cohort) treated with Dynole 34-2, VXL or combination therapy. The period of administration is indicated in light grey. **g,** Leukemia growth delay (LGD) in recipients injected with *Lmo2*^{Tg} primary leukemias treated with Dynole 34-2 as a single agent, VXL and combination therapy. Median is indicated for each condition (bar) with Mean ± SEM indicated for each primary T-ALL, 2-way ANOVA with Tukey's correction test; $^{*}P<0.05$, $^{***}P<0.001$ as compared to vehicle; $^{\#\#\#}P<0.001$ compared to VXL.

a

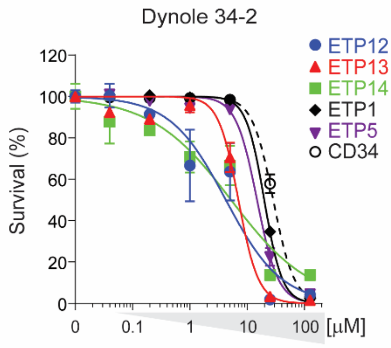

b

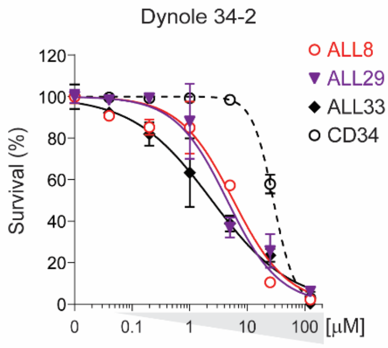

ETP-ALL	Genetic lesions in signalling genes	LC_{50} [μM]
ETP1	*DNM2* K557_K558 > K, *JAK3* M511I	19.9
ETP5	*PTPN11* A72V	14.9
ETP8	*JAK1* E1012 > EK, *SH2B3* V65A, I257T	N/A
ETP12	*NOTCH1* S2492*, *SH2B3* SV	4.26
ETP13	None	7.05
ETP14	*JAK1* S703I, *PTEN* SV	5.25

T-ALL	Genetic lesions in signalling genes	LC_{50} [μM]
ALL8	*FBXW7* R465C	5.76
ALL29	*NOTCH1* P2514fs	4.51
ALL16	*NOTCH1* L1600P	N/A
ALL33	*NOTCH1* N325Y	2.43
ALL31	*FBXW7* T15_R16 > L	N/A

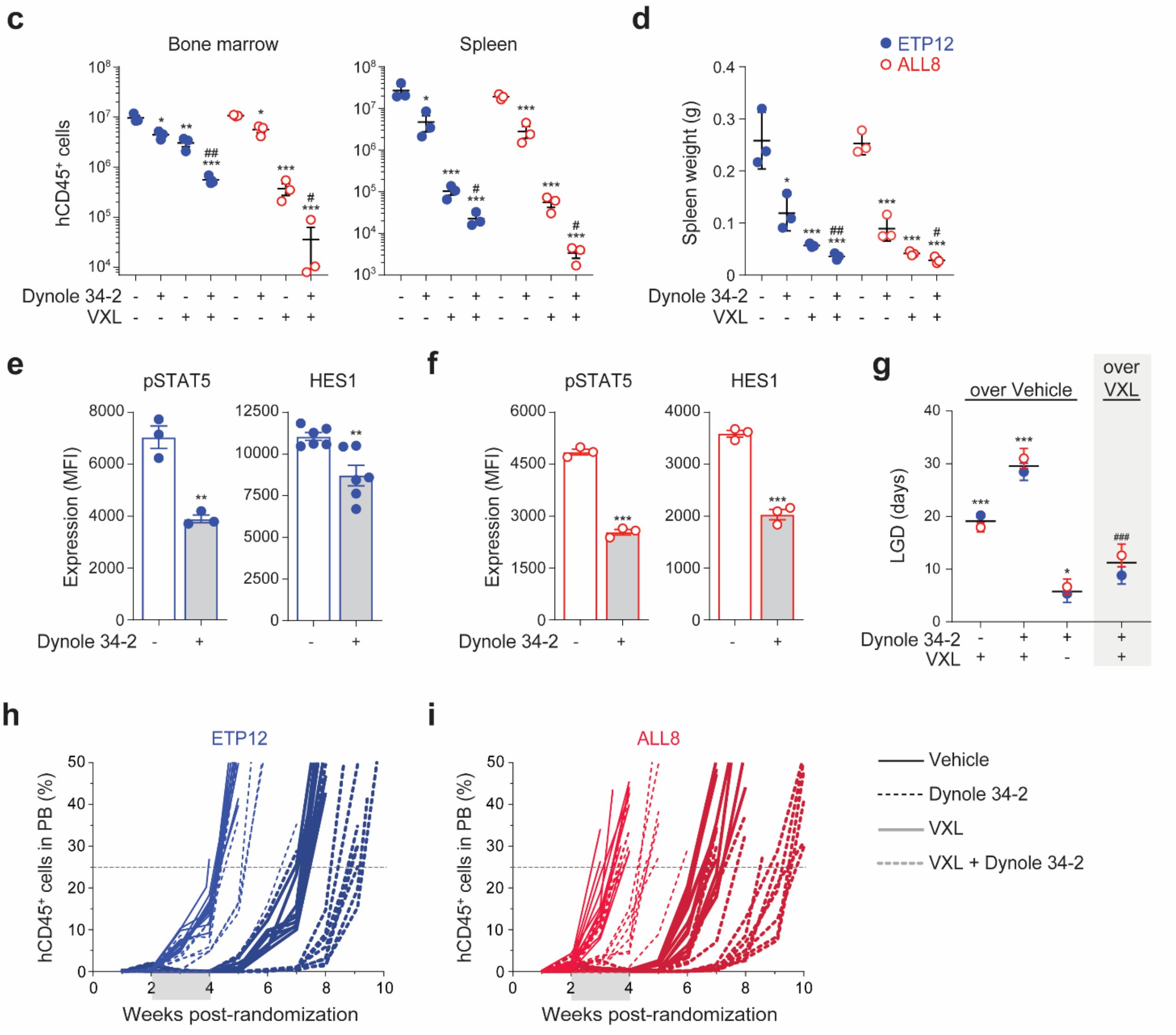

Supplementary Figure 7. Efficacy of Dynole 34-2 for human T-ALL. a, b, Relative viability (%) of patient-derived (**a**) ETP-ALL[1] and (**b**) T-ALL[2] xenografts treated with increasing concentration of Dynole 34-2 for 48 hours. Viability was normalized to vehicle-treated cells with experiments performed in duplicates. Human CD34-positive (CD34+) hematopoietic stem and progenitors from healthy donors (N=3) were used as controls. Mutations of growth factor-induced signalling pathways and median lethal concentration (LC_{50}) of Dynole 34-2 for each patient-derived xenografts are displayed. The amino-acid position for each mutation is shown, * means frameshift. Full characterisation of these patient-derived ETP-ALL and T-ALL xenografts is provided in **Table 1**. Patient-derived T-ALL samples can be accessed upon request, following approval by the Human Research Ethics Committee of the University of New South Wales (UNSW), and the Alfred Health Human Ethics Committee. **c, d**, Absolute number of patient-derived ($hCD45^+$) leukemic cells in the bone marrow and spleen (**c**), as well as the spleen weight (**d**), in recipients injected with ETP-ALL (blue circles) and ALL8 (red open circles) 24 hours after the last dose of drugs was administered. Mean ± SEM, Student's *t*-test; $^*P<0.05$, $^{***}P<0.001$ as compared to vehicle; $^{\#}P<0.05$ compared to VXL. **e, f,** Levels of pSTAT5 and HES1 in ETP12 (**e**) and ALL8 (**f**) patient-derived cells, harvested from the bone marrow of recipients 24 hours after the last administration of Dynole 34-2, assessed by flow cytometry. Mean Fluorescence Intensity (MFI) ± SEM, Student's *t*-test; $^{**}P<0.01$, $^{***}P<0.001$ as compared to vehicle. **g,** Leukemia growth delay (LGD) in recipients injected with ETP12 (blue circles) and ALL8 (red open circles) treated with Dynole 34-2 as a single agent, VXL and combination therapy. Median is indicated (bar) with Mean ± SEM indicated for ETP12 and ALL8, 2-way ANOVA with Tukey's correction test; $^*P<0.05$, $^{***}P<0.001$ as compared to vehicle; $^{\#\#\#}P<0.001$ compared to VXL. **h, i**, Proportion of patient-derived leukemic cells (%$hCD45^+$) in the peripheral blood recipients injected with ETP12 (**h**) and ALL8 (**i**), treated with Dynole 34-2, VXL or combination therapy. The period of administration is indicated in light grey.

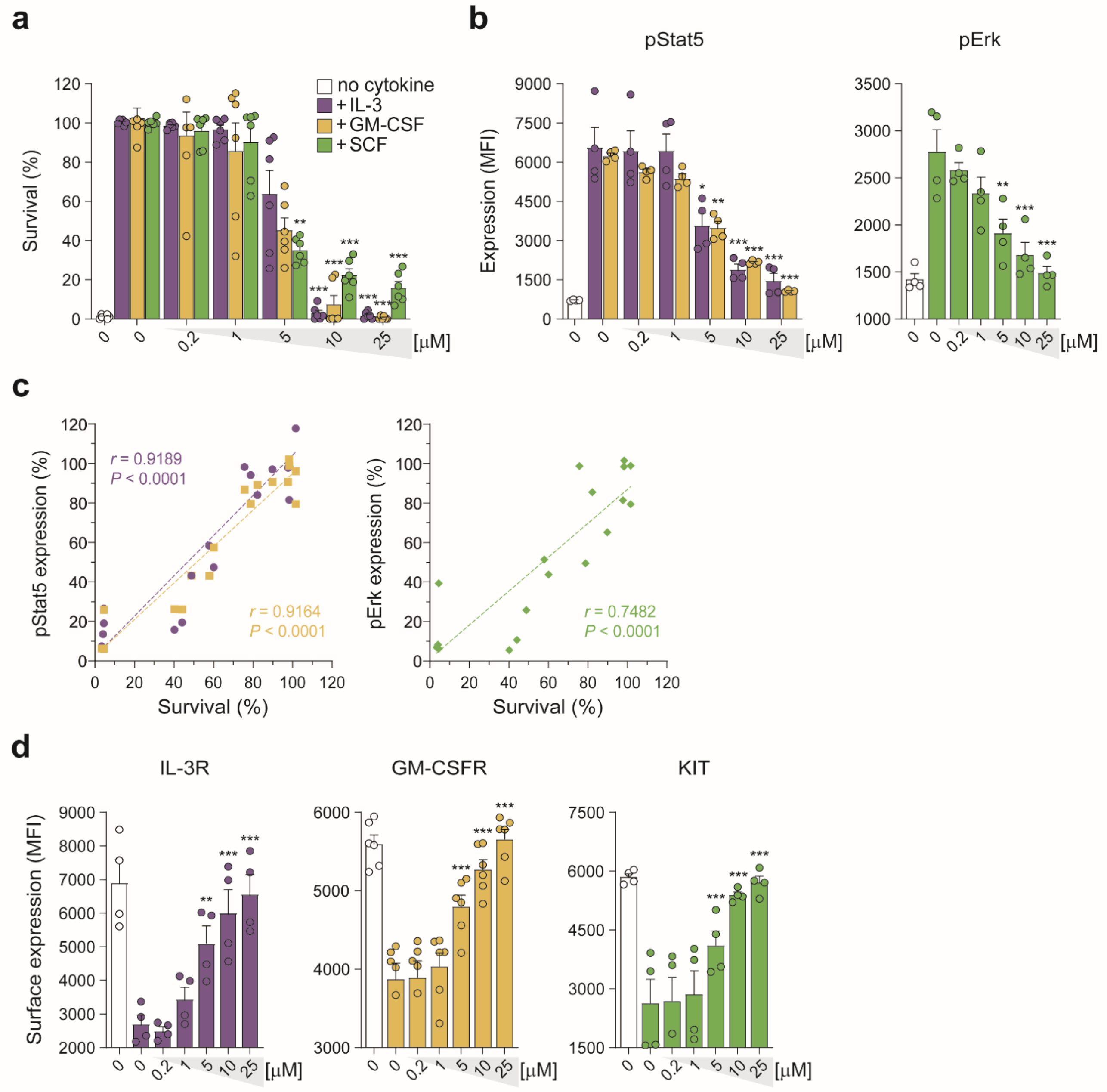

Supplementary Figure 8. ***In vitro* treatment of BaF3-SGM3R cells with Dynole 34-2. a,** Relative Survival of cytokine-dependent BaF3-SGM3R cells treated with Dynole 34-2 in the presence of cytokines for 48 hours, as indicated (+IL-3, purple; +GM-CSF, yellow; +SCF, green). Viability was normalized to cells treated with vehicle (%DMSO). Mean ± SD from 3 biological replicates. Student's *t*-test **P*<0.05, ***P*<0.01, ****P*<0.001 compared to vehicle. Basal levels of live cells were measured in BaF3-SGM3R cells cultured without cytokines (white bar). **b,** Levels of activated Stat5 (pStat5) in BaF3-SGM3R cells stimulated with IL-3 (purple bars) and GM-CSF (yellow bars), and SCF-induced phospho-Erk (green bars), treated with increasing doses of Dynole 34-2 assessed by flow cytometry.

Mean fluorescence intensity (MFI) ± SD of n=3 biological replicates are shown (**P*<0.05, ***P*<0.01, ****P*<0.001 compared to vehicle). Basal levels of pStat5 and pErk were measured in unstimulated BaF3-SGM3R cells (white bar). **c,** Correlative studies between the relative levels of pStat5 (left) or pErk (right) and the relative survival of BaF3-SGM3R cells treated Dynole 34-2. Pearson correlation coefficient *r* is indicated. Student's *t*-test. Levels of pStat5 in presence of IL-3 (purple) or GM-CSF (yellow), pErk in presence of SCF (green) and viability was normalized to cells treated with vehicle (%DMSO). **d,** Surface expression of receptors for IL-3 (IL-3R), GM-CSF (GM-CSFR) and SCF (KIT) in BaF3-SGM3R cells treated with Dynole 34-2. MFI ± SD of n=3 biological replicates are shown (***P*<0.01, ****P*<0.001 compared to vehicle). Baseline measured in unstimulated BaF3-SGM3R cells (white bar).

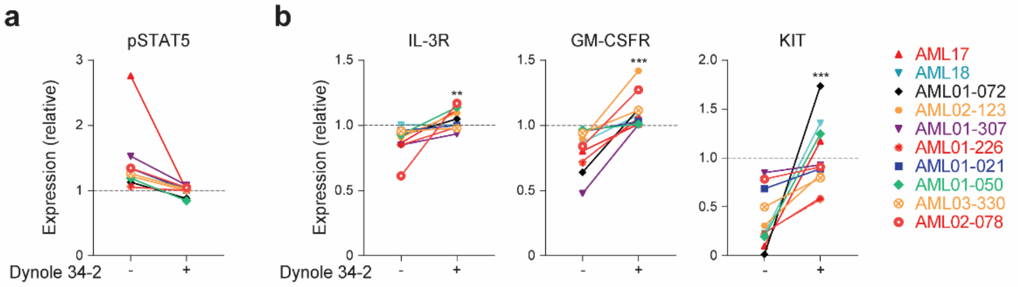

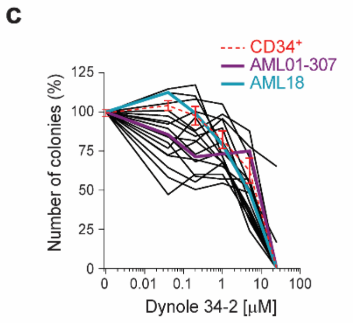

d

AML	Genetic lesions in signalling genes	LC_{50} [μM]
AML01-152	None	31.4
AML01-012	*KIT* D816V	19.6
AML04-155	None	12.5
AML01-186	None	9.5
AML03-124	None	7.8
AML01-021	*JAK2* V617F	7.4
AML01-307	*FLT3*-ITD	6.7
AML01-072	*FLT3*-ITD	6.4
AML18	*KRAS* G12A	4.3
AML01-064	None	4.0
AML02-012	*NRAS* G12S	3.6
AML01-226	*FLT3*-ITD	3.6
AML01-050	None	3.3
AML17	*FLT3*-ITD	2.6
AML01-016	*FLT3* D835Y, *NRAS* G12D	2.2
AML01-318	None	1.3
AML01-044	*FLT3*-ITD	1.3
AML04-015	*NRAS* G12A, *KIT* D816V, *FLT3* D835H	0.98
AML03-330	None	0.91
AML02-123	None	0.39

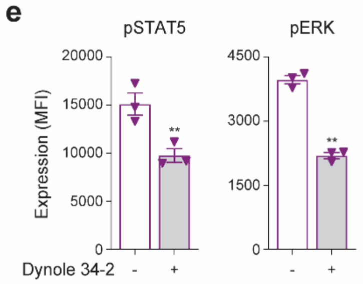

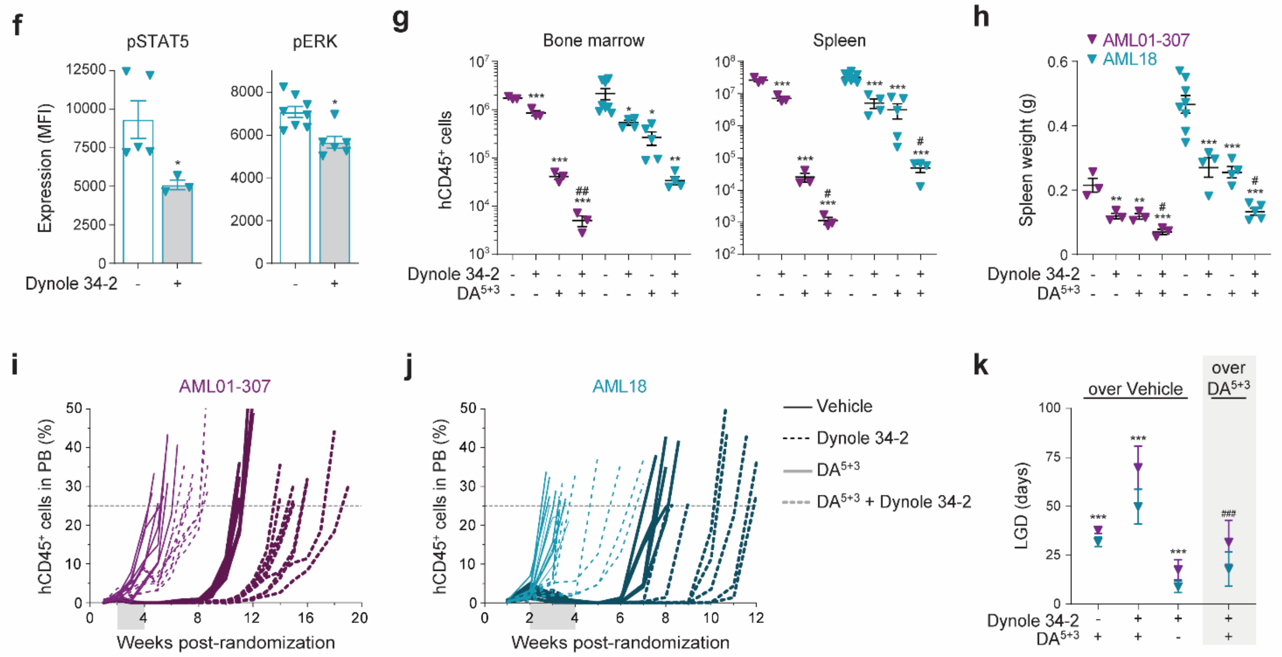

Supplementary Figure 9. Efficacy of Dynole 34-2 for human AML. a, Relative levels of activated STAT5 (pSTAT5) in patient-derived AML cells treated with vehicle and Dynole 34-2, after *in vitro*

stimulation with GM-CSF. **b,** Surface expression of receptors for IL-3 (IL-3R), GM-CSF (GM-CSFR) and SCF (KIT) treated with Dynole 34-2 assessed by flow cytometry. Levels in unstimulated xenograft cells treated with vehicle were used as control, and reported as 1 (dashed line). Mean Fluorescence Intensity (MFI) ± SD on grouped samples, 2-way ANOVA with Bonferroni correction test; $^{**}P<0.01$ and $^{***}P<0.001$ as compared to vehicle. **c,** Clonogenic assay of patient-derived AML cells cultured in the presence of either vehicle or Dynole 34-2 for 10 days. AML01-307 is depicted in solid purple, AML18 in solid turquoise, and other AML samples with individual black lines. Human CD34-positive (red dashed line; CD34+) hematopoietic stem and progenitor cells from healthy donors (N=3) were used as controls. **d,** Mutations of growth factor-induced signalling pathways and median lethal concentration (LC_{50}) of Dynole 34-2 for patient-derived AML xenografts tested for *in vitro* stimulation assays. The amino-acid position for each mutation is shown, * means frameshift. Full characterisation of these human AML samples is provided in **Table 2**. Patient-derived AML samples can be accessed upon request, following approval by the Human Research Ethics Committee of the University of New South Wales (UNSW), and the Alfred Health Human Ethics Committee. **e, f,** Levels of pSTAT5 and pERK in AML01-307 (**e**) and AML18 (**f**) patient-derived cells, harvested from the bone marrow of recipients 24 hours after the last administration of Dynole 34-2, assessed by flow cytometry. MFI ± SEM, Student's *t*-test; $^{*}P<0.05$, $^{**}P<0.01$ as compared to vehicle. **g, h,** Absolute number of patient-derived ($hCD45^{+}$) leukemic cells in the bone marrow and spleen (**g**), as well as the spleen weight (**h**), in recipients injected with AML01-307 (purple triangle) and AML18 (turquoise triangles) cells, enumerated 24 hours after the administration of the last dose of drugs. Mean ± SEM, Student's *t*-test; $^{**}P<0.01$, $^{***}P<0.001$ as compared to vehicle; $^{\#}P<0.05$, $^{\#\#}P<0.01$ compared to DA^{5+3}. **i, j**, Proportion of patient-derived leukemic cells (%$hCD45^{+}$) in the peripheral blood recipients injected with AML01-307 (**i**) and AML18 (**j**), treated with Dynole 34-2, DA^{5+3} or combination therapy. The period of administration is indicated in light grey. **k,** Leukemia growth delay (LGD) in recipients injected with AML01-307 (purple triangle) and AML18 (turquoise triangles) cells treated with Dynole 34-2 as a single agent, DA^{5+3} and combination therapy. Median is indicated (bar) with Mean ± SEM, 2-way ANOVA with Tukey's correction test; $^{***}P<0.001$ as compared to vehicle; $^{\#\#\#}P<0.001$ compared to DA^{5+3}.

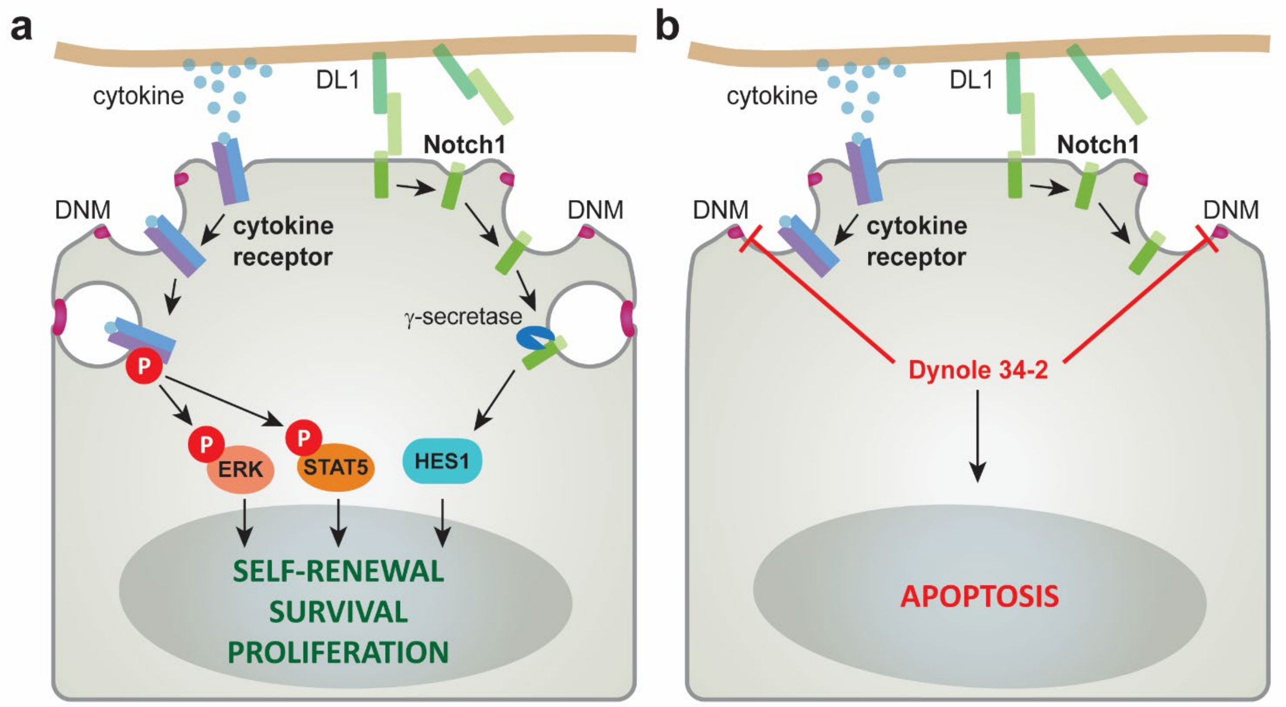

Supplementary Figure 10. Dynamin inhibitors target relapse-inducing cells in acute leukemia. **a,** Schematic model of the transduction of niche-induced signalling pathways in acute leukemia. Stimulation by cytokines or Notch1 ligand Delta-like 1 (DL1) triggers the Dynamin-dependent endocytosis (DDE) of ligand-bound receptors of cytokines (IL-7, IL-3, GM-CSF and SCF) and Notch1, leading to the downstream activation of canonical effectors (e.g. pSTAT5 and pERK) of these signalling pathways and expression HES1 (the canonical effector of Notch1), which are responsible for self-renewal, survival and proliferation of relapse-inducing cells. **b,** Inhibition of DDE by Dynole 34-2 prevents the internalization of ligand-bound receptors and downstream activation of canonical effectors, leading to apoptosis of relapse-inducing cells. DNM = Dynamin, P = phosphorylation.

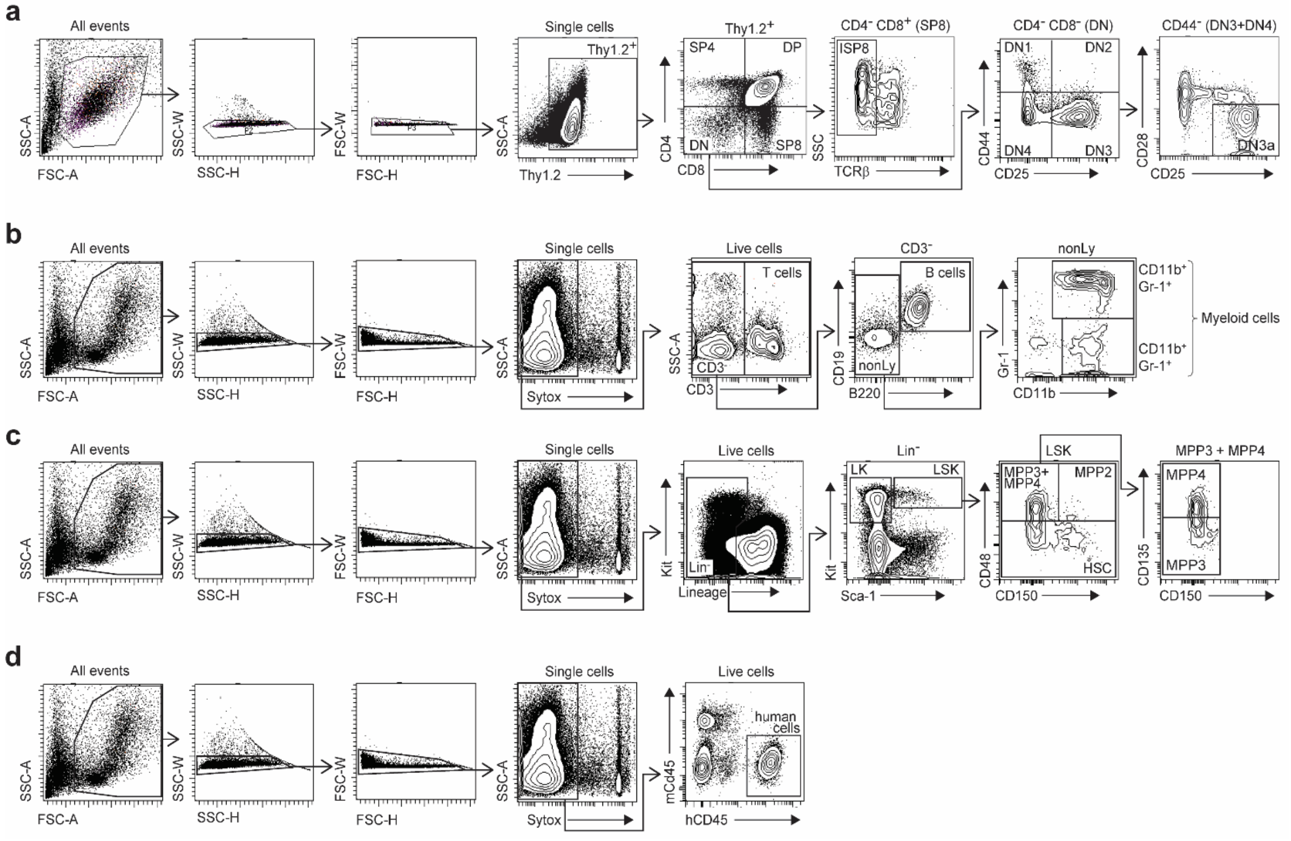

Supplementary Figure 11. Gating strategies for flow cytometric analyses. a, T-cell population analyses were performed using the gating strategy represented, as previously described[3, 4]. **b,** Flow cytometric analysis of mature hematopoietic populations (lineage$^+$) were performed using CD3 (T cells), CD19 and B220 (B cells), CD11b and Gr-1 (Myeloid cells) surface markers. NonLy = non-lymphoid cells. **c,** Hematopoietic and progenitor cell (HSPC) population analyses were performed using the gating strategy represented below, as previously described[5]. Lin$^-$ = lineage negative; LK = Lin$^-$ Kit$^+$; LSK = Lin$^-$ Sca-1$^+$ Kit$^+$ cells; MPP = multipotent progenitors. **d,** Flow cytometric analysis of patient-derived xenografts (PDX) using murine (mCd45) and human CD45 (hCD45) surface markers.

Supplementary References

1. Maude SL*, et al.* Efficacy of JAK/STAT pathway inhibition in murine xenograft models of early T-cell precursor (ETP) acute lymphoblastic leukemia. *Blood* **125**, 1759-1767 (2015).

2. Moradi Manesh D*, et al.* AKR1C3 is a biomarker of sensitivity to PR-104 in preclinical models of T-cell acute lymphoblastic leukemia. *Blood* **126**, 1193-1202 (2015).

3. Tremblay CS*, et al.* Loss-of-function mutations of Dynamin 2 promote T-ALL by enhancing IL-7 signalling. *Leukemia*, (2016).

4. Tremblay CS*, et al.* Restricted cell cycle is essential for clonal evolution and therapeutic resistance of pre-leukemic stem cells. *Nature communications* **9**, 3535 (2018).

5. Pietras EM*, et al.* Functionally Distinct Subsets of Lineage-Biased Multipotent Progenitors Control Blood Production in Normal and Regenerative Conditions. *Cell stem cell* **17**, 35-46 (2015).
