# Supplementary material for: Small molecule inhibition of Dynamin-dependent endocytosis targets multiple niche signals and impairs leukemia stem cells
Source: Nat Commun. 2020 Dec 4;11:6211. doi: 10.1038/s41467-020-20091-6 (PMC7719179; doi:10.1038/s41467-020-20091-6)
Supplement: Supplementary file 3 — Reporting summary [file 41467_2020_20091_MOESM3_ESM.pdf]

nature research

Corresponding author(s): Cedric S Tremblay

Last updated by author(s): Oct 27, 2020

Reporting Summary

Nature Research wishes to improve the reproducibility of the work that we publish. This form provides structure for consistency and transparency in reporting. For further information on Nature Research policies, see our Editorial Policies and the Editorial Policy Checklist.

Statistics

For all statistical analyses, confirm that the following items are present in the figure legend, table legend, main text, or Methods section.

n/a	Confirmed	
☐	☒	The exact sample size (*n*) for each experimental group/condition, given as a discrete number and unit of measurement
☐	☒	A statement on whether measurements were taken from distinct samples or whether the same sample was measured repeatedly
☐	☒	The statistical test(s) used AND whether they are one- or two-sided<br>*Only common tests should be described solely by name; describe more complex techniques in the Methods section.*
☐	☒	A description of all covariates tested
☐	☒	A description of any assumptions or corrections, such as tests of normality and adjustment for multiple comparisons
☐	☒	A full description of the statistical parameters including central tendency (e.g. means) or other basic estimates (e.g. regression coefficient) AND variation (e.g. standard deviation) or associated estimates of uncertainty (e.g. confidence intervals)
☐	☒	For null hypothesis testing, the test statistic (e.g. *F*, *t*, *r*) with confidence intervals, effect sizes, degrees of freedom and *P* value noted<br>*Give P values as exact values whenever suitable.*
☒	☐	For Bayesian analysis, information on the choice of priors and Markov chain Monte Carlo settings
☐	☒	For hierarchical and complex designs, identification of the appropriate level for tests and full reporting of outcomes
☐	☒	Estimates of effect sizes (e.g. Cohen's *d*, Pearson's *r*), indicating how they were calculated

Our web collection on statistics for biologists contains articles on many of the points above.

Software and code

Policy information about availability of computer code

Data collection	All relevant raw data have been included in the Source Data file
Data analysis	N/A

For manuscripts utilizing custom algorithms or software that are central to the research but not yet described in published literature, software must be made available to editors and reviewers. We strongly encourage code deposition in a community repository (e.g. GitHub). See the Nature Research guidelines for submitting code & software for further information.

Data

Policy information about availability of data

All manuscripts must include a data availability statement. This statement should provide the following information, where applicable:

- Accession codes, unique identifiers, or web links for publicly available datasets
- A list of figures that have associated raw data
- A description of any restrictions on data availability

All data generated or analyzed during this study are included in this article (and its supplementary information files). The relevant data that support the findings of this study are available from the corresponding author upon reasonable request.

Field-specific reporting

Life sciences study design

All studies must disclose on these points even when the disclosure is negative.

Sample size	Sample size for each experiment were determined using the following equation: N=2s2((Za/2+Zb)2) / (un-uo)2 where standard deviation (s), observed difference (un-uo), and significance of p <0.05 (Za/2+Zb)2 were established based upon previous experiments.
Data exclusions	N/A
Replication	All experiments were replicated, and the data presented was generated from independent experiments
Randomization	All in vivo studies were performed in randomized mice, with similar distributions for each cohorts
Blinding	All in vivo experiments were blinded, as they were performed and monitored by different researchers, with samples analysed by a another investigator.

Reporting for specific materials, systems and methods

We require information from authors about some types of materials, experimental systems and methods used in many studies. Here, indicate whether each material, system or method listed is relevant to your study. If you are not sure if a list item applies to your research, read the appropriate section before selecting a response.

Materials & experimental systems

n/a	Involved in the study	
☐	☒	Antibodies
☐	☒	Eukaryotic cell lines
☒	☐	Palaeontology and archaeology
☐	☒	Animals and other organisms
☒	☐	Human research participants
☒	☐	Clinical data
☒	☐	Dual use research of concern

Methods

n/a	Involved in the study	
☒	☐	ChIP-seq
☐	☒	Flow cytometry
☒	☐	MRI-based neuroimaging

Antibodies

Antibodies used	For most antibodies, only the clones are indicated, as they were used with different fluorophores, and ordered through different lot. When only one set has been used, all informations were listed (ie, clone, catalog number, lot number).<br>AnnexinV (#556420), hCD45 (HI30; #557748), hNOTCH1 (MHN1-519), cleaved CASPASE 3 (C92-605), cytoplasmic CD3 (HCHT1), CD4 (RM4-5), CD8 (53-6.7), CD25 (PC61.5), CD44 (IM7), CD45.1 (A20), CD45.2 (104), Thy1.2 (53-2.1), TCRβ (H57-597), CD117 (ACK45), CD135 (A2F10.1), CD150 (TC15-12F12.2; #562373), B220 (RA3-6B2; #563708), CD3 (145-2C11; #563004), CD19 (ID3; #557655), Gr-1 (RB6-8C5; #563299 and #565033), CD11b (M1/70; #563015 and #560455), Ter119 (Ter119; #565523 and #557909), Sca-1 (D7; #558160) - BD Pharmingen antibodies (BD Australia, North Ryde NSW, Australia), different lots<br>NOTCH1 (22E5) - eBioscience antibody (eBioscience, Invitrogen), different lots<br>hKIT (A3C6E2), hCD127 (A019D5), CD28 (E18) and CD127 (A7R34) - BioLegend antibodies (Australian Biosearch, Balcatta WA, Australia), different lots<br>HES1 (D6PU; #11988), phospho-STAT5 (Tyr694; #9359), phospho-p38 MAPK (Thr180/Tyr182; #4511), phospho-p44/42 (Erk1/2; #9102), phospho-S6 (Ser235/236; #4856) and phospho-Akt (Ser473; #9271) - Cell Signalling purified antibodies (Genesearch Pty Ltd, Arundel QLD, Australia), several lots<br>donkey Alexa Fluor 488-conjugated anti-rabbit secondary antibody (A-21206, lot #1910751, Molecular Probes, Invitrogen) or goat Alexa Fluor 546-conjugated anti-rabbit secondary antibody (A-11010, lot #1904467, Molecular Probes, Invitrogen)
Validation	All antibodies used were validated in the laboratory prior to performing research.

Eukaryotic cell lines

Policy information about cell lines

Cell line source(s)	Ba/F3: kindly provided by Ms Sandra Mifsud from The Walter and Eliza Hall Institute of Medical Research
Authentication	Cell line was not authenticated
Mycoplasma contamination	Tested negative for Mycoplasma, and were regularly tested
Commonly misidentified lines (See ICLAC register)	N/A

Animals and other organisms

Policy information about studies involving animals; ARRIVE guidelines recommended for reporting animal research

Laboratory animals	CD2-Lmo2 (Lmo2Tg) mouse model. Mice cohorts were generated by cross-breeding with the Lck-Notch1-IC9 (N1-ICDTg) generously given by T. Hoang 83, the Lck-STAT5b-CA (STAT5-CATg)85 kindly given by M.P. McCormack, the Mx1-Cre transgenic (Mx) and the ROSA26-YFP Cre-reporter (YFP) mouse strains. All mouse lines were backcrossed onto a C57BL/6J background for 10 generations and maintained in pathogen-free conditions according to institutional animal care guidelines.<br>PDX studies were performed with nonobese diabetic/severe combined immunodeficient (NOD.Cg-Prkdcscid, also termed NOD/SCID) or NOD/SCID/Il2rgtm1wjl/SzJ (NSG) mice (T-ALL) or nonobese diabetic/severe immunodeficient (NOD.Cg-Rag1tm1MomIl2rgtm1wjl/SzJ, also termed NRG) mice expressing human interleukin-3 (IL-3), human granulocyte/macrophage-stimulating factor (GM-CSF) and human SCF from the SGM3 triple transgene (NRGS). Both males and females were used.
Wild animals	N/A
Field-collected samples	N/A
Ethics oversight	All experiments were pre-approved by the Human Research Ethics Committee of the University of New South Wales (UNSW), the UNSW Animal Care and Ethics Committee, the Alfred Health Human Ethics Committee and the AMREP Animal Ethics Committee. All bone marrow or peripheral blood samples from patients with AML were collected after informed consent, in accordance with guidelines approved by the Human Research Ethics Committee (HREC) of Alfred Health.

Note that full information on the approval of the study protocol must also be provided in the manuscript.

Flow Cytometry

Plots

Confirm that:

- [x] The axis labels state the marker and fluorochrome used (e.g. CD4-FITC).
- [x] The axis scales are clearly visible. Include numbers along axes only for bottom left plot of group (a 'group' is an analysis of identical markers).
- [x] All plots are contour plots with outliers or pseudocolor plots.
- [x] A numerical value for number of cells or percentage (with statistics) is provided.

Methodology

Sample preparation	Phosphoflow analysis was performed as previously described. Briefly, 2x106 cells stained for surface markers were subsequently resuspended into PBS 1X + 4% v/v PFA, and incubated for 10 minutes at 4oC for fixation. Fixed cells were washed twice using PBS 1X, then resuspended in pre-chilled at -20oC Perm Buffer III (BD Pharmingen) and incubated 30 minutes at 4oC for permeabilization. Cells were washed twice using PBS 1X, and incubated overnight at 4oC in PBS 1X + 2% v/v FCS with primary anti-phospho antibodies or isotype control. For intracellular HES1 detection, cells stained for surface markers were subsequently fixed and permeabilized using the BD Cytofix/CytopermTM Kit and incubated overnight at 4oC in PBS 1X + 2% v/v FCS with a purified antibody against HES1 (D6PU; #11988, Cell Signalling) or isotype control. After staining with primary antibodies for phosphoflow or intracellular detection, cells were incubated in permeabilization buffer with the relevant fluorophore-complexed secondary antibody for 1 h on ice, and washed twice in PBS 1X.
Instrument	FACS analysis was performed using LSRII and LSR Fortessa cytometers. Cell sorting was performed with a FACSAria or BD Influx.
Software	FACS Diva
Cell population abundance	DN1 (0.1%), DN3a (1%), DN4 (1%), DP (80%) of Thy1.2-positive cells, on average, in wild-type mice. As previously described in previous publications by this author. Other analyses also list relevant populations.
Gating strategy	Cells are analysed on SSC-A vs FSC-A, then doublets are excluded using the SSC-H vs SSC-W and FSC-H vs FSC-W parameters. Gating strategy for thymocytes is provided in Figure 5a, and extensively described in the Supplementary Methods section.

- [x] Tick this box to confirm that a figure exemplifying the gating strategy is provided in the Supplementary Information.
